# Turning in Circles: Understanding Manual Wheelchair Use Towards Developing User-Friendly Steering Systems

**DOI:** 10.3389/fbioe.2022.831528

**Published:** 2022-02-17

**Authors:** Reto Togni, Andrea Kilchenmann, Alba Proffe, Joel Mullarkey, László Demkó, William R. Taylor, Roland Zemp

**Affiliations:** ^1^ Laboratory for Movement Biomechanics, Institute for Biomechanics, Zurich, Switzerland; ^2^ Spinal Cord Injury Research Center, University Hospital Balgrist, Zurich, Switzerland

**Keywords:** manual wheelchair use, turn detection, wearable sensors, activities of daily living, accelerometer, rehabilitation, movement patterns, mobility

## Abstract

For people with physical disabilities, manual wheelchairs are essential enablers of mobility, participation in society, and a healthy lifestyle. Their most general design offers great flexibility and direct feedback, but has been described to be inefficient and demands good coordination of the upper extremities while critically influencing users’ actions. Multiple research groups have used Inertial Measurement Units (IMUs) to quantify physical activities in wheelchairs arguing that knowledge over behavioural patterns in manual wheelchair usage can guide technological development and improved designs. The present study investigates turning behaviour among fulltime wheelchair users, laying the foundation of the development of novel steering systems that allow directing kinetic energy by means other than braking. Three wearable sensors were installed on the wheelchairs of 14 individuals for tracking movement over an entire week. During detected “moving windows”, phases where the velocities of the two rear wheels differed by more than 0.05 m/s were considered as turns. Kinematic characteristics for both turns-on-the-spot as well as for moving turns were then derived from the previously reconstructed wheeled path. For the grand total of 334 km of recorded wheelchair movement, a turn was detected every 3.6 m, which equates to about 900 turns per day on average and shows that changing and adjusting direction is fundamental in wheelchair practice. For moving turns, a median turning radius of 1.09 m and a median turning angle of 39° were found. With a median of 89°, typical turning angles were considerably larger for turns-on-the-spot, which accounted for roughly a quarter of the recognised turns and often started from a standstill. These results suggest that a frequent pattern in daily wheelchair usage is to initiate movement with an orienting turn-on-the-spot, and cover distances with short, straightforward sections while adjusting direction in small and tight moving turns. As large bends often require simultaneous pushing and breaking, this is, perhaps, the result of users intuitively optimising energy efficiency, but more research is needed to understand how the design of the assistive devices implicitly directs users’ movement.

## Introduction

According to the World Health Organisation, approximately 1% of the population with musculoskeletal impairments or disabilities, or worldwide more than 65 million people rely on a wheelchair for mobility (WHO, 2008). The same report highlights the critical role of an adequate assistive device for people with disabilities to become mobile, participate fully and, above all, remain healthy, which emphasises the multifaceted relationship between the wheelchair and the manifold activities users engage in. While wheelchairs are central to users’ independence, opportunities, and health, almost 90% are surprisingly simple, mechanical devices (van der Woude et al., 2001).

Wheelchairs generally use four independent wheels and are push-rim controlled, meaning that hand-rims on the large rear wheels act as the interface for propelling and navigating the system: pushing forward on both sides simultaneously results in a forward motion, while braking on one side causes a turn to the respective direction. Although offering great flexibility and direct visual, proprioceptive and kinaesthetic feedback (van der Woude et al., 2006), such a configuration has been described to render wheelchair ambulation a challenging, frustrating and hugely inefficient process (Reid et al., 1990; Lee Kirby et al., 1999) that requires the constant use of both hands, a specific set of skills and good coordination of the upper extremities. Concurrently, a strong ability to effectively and fluently ambulate a manual wheelchair has been correlated with a general increase in activity, participation and quality of life (Hosseini et al., 2012; Kirby et al., 2016) while, inversely, insufficient skills or training are a limiting factor, and may lead to an increased risk for accidents or injuries (Kirby et al., 2016). Overall, the fundamental principle of manual wheelchair-user-interaction appears to impose requirements on a user’s physical abilities and shape their activities. In a similar argumentation that dates back to 1995, Simmons and others (Simmons et al., 1995) suggested that improving the user-friendliness of wheelchairs might result in increased freedom of movement for users. Twenty years later, Medola and others (Medola et al., 2014) concluded that even small details in a wheelchair’s configuration can affect not only a user’s overall mobility but also manoeuvre and propulsion patterns. Indeed, there are several ways in which the design of a wheelchair directly influences a user’s actions. As a plain example, wheelchair users are often required to take a detour to avoid steps or, on a more fine-grained level, might find themselves adapting to topographical features, as even slightly uneven surfaces cause “veering off” in conventional wheelchair designs (Storch, 2015).

This study aimed to lay the foundations for introducing novel interaction-principles in the design of manual wheelchairs based on the concept of steering systems that allow directing kinetic energy by means other than braking. Multiple groups have argued that knowledge of wheelchair usage in daily life is an invaluable resource to the design, engineering, and manufacture of new, possibly improved models (Sonenblum et al., 2012a; Sonenblum et al., 2012b; Medola et al., 2014; Cooper and Cooper, 2019; Misch et al., 2020). Here, the objective assessment of turning behaviour among users of conventional wheelchairs is a key step towards understanding the spatial constraints faced during everyday life and the resulting manoeuvrability requirements for new designs. Such knowledge further provides a basis for estimating the effects of changing fundamental principles in the wheelchair-user combination. Importantly, any major changes in wheelchair design will likely only find acceptance if they integrate well with existing movement patterns, especially as many users, particularly with chronic health conditions, might have practiced techniques and established routines. Understanding kinematics, occurrences and patterns in wheelchair turning is therefore critical to the further development of steering wheelchairs.

Towards improving rehabilitation process, different portable measurement systems have been proposed for tracking wheelchair user’s activity, for example by monitoring and promoting active sitting (Sonenblum et al., 2016; Hubli et al., 2021). Similarly, the properties of physical activity during daily living of wheelchair users have been investigated (Tolerico et al., 2007; Cooper et al., 2008; Karmarkar et al., 2010; Oyster et al., 2011; Sonenblum et al., 2012a; Hiremath et al., 2013). Multiple research groups have used inertial measurement units (IMUs) for long-term measurements to obtain information on distance travelled, time spent moving, or the velocity of self-propelled travel (Sonenblum et al., 2012c; van der Slikke et al., 2015; Popp et al., 2016; Popp et al., 2018; Schneider et al., 2018). Sonenblum and others (Sonenblum et al., 2012c) described the daily wheelchair usage in “bouts of mobility”, which were defined as continuous moving segments with a minimum duration of 5 s. Their study provides insight into the moving behaviour of manual wheelchair users and shows that most bouts are short and slow with a median duration of 21s and velocity of 0.43 m/s. Other studies focused on wheelchair kinematics (van der Slikke et al., 2015), the evaluation of physical activity levels (Popp et al., 2016; Tsang et al., 2016; Popp et al., 2018; Schneider et al., 2018), or on the characteristics of movement during wheelchair sports (van der Slikke et al., 2015; van der Slikke et al., 2016).

Interestingly, rotation behaviour is developing as an important parameter in neuropsychological studies, where the number of left/right turns are thought to be indicative of laterality or rotation biases (Schaeffer, 1928; Bracha et al., 1993; Souman et al., 2009; Leuenberger et al., 2015). However, to the best of our knowledge, no study has attempted to describe turning behaviour of wheelchair users in general, or quantify turning radii specifically. Knowledge of normal turning characteristics is critical, before such measures can represent biomarkers for evaluating neurological deficits or quantifying pathological performance, but also for supporting the development of novel steering systems that meet user’s needs. This study therefore builds on a body of research describing the daily activities of wheelchair users based on IMU data, but focuses on identifying and describing turns and manoeuvres. Details about number of turns per day, turning radii, angles and velocities, as well as the detection of common turning patterns, can provide a unique insight into *how* people negotiate spaces and move from one place to another.

## Methods

### Participants

Individuals who have relied on a manual wheelchair for mobility for a duration of at least 6 months before the measurements were included in this study. Participants were required to be at least 18 years old, active wheelchair users that are able to propel their wheelchairs independently. Exclusion criteria included suffering from an acute injury, such as a pressure ulcer or shoulder injury, as well as untreated mental illnesses such as depression. The study was approved by the ethics committee of ETH Zurich (EK 2020-N-04), and all participants provided written informed consent prior to the start of measurements.

### Data Collection

For this study, a set of three ZurichMOVE (Leuenberger and Gassert, 2011) sensors was used for all measurements, each recording data from the 3-axis accelerometer with a range of ±16 g, and the 3-axis gyroscope with a range of ±2000°/s (both with a resolution of 16 bit), as well as quaternions from the onboard motion processor (9-axis on-chip MotionFusion of MPU-9250, InvenSense, San Jose, CA, United States). The sensors were synchronised while the data was stored locally on inbuilt SD-cards. Sampling frequency was set to 50 Hz. The sensors were adapted with a large battery to allow for consecutive measurements of 7 days without recharging–that is, without any manipulation - hence enabling the reliable capture of variable physical activities of wheelchair users (Schneider et al., 2018).

**FIGURE 1:**
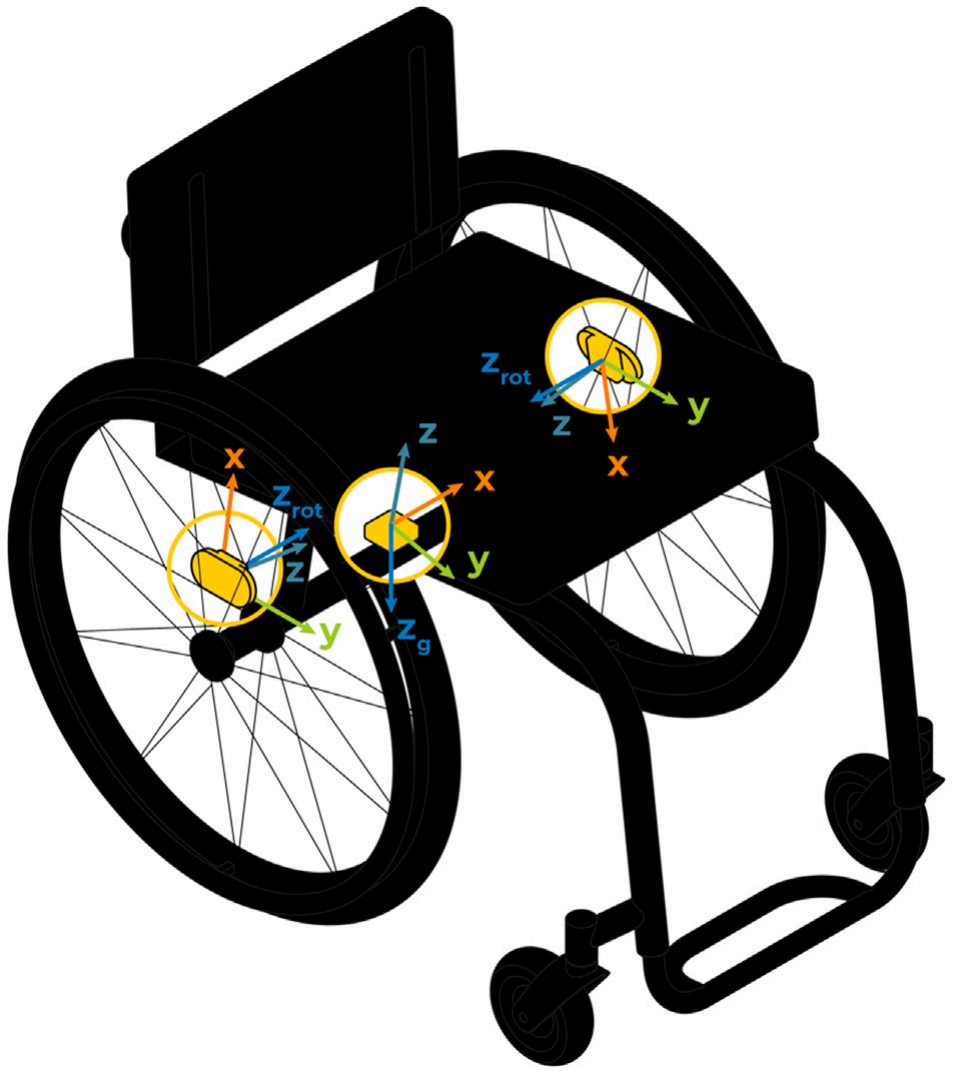
A set of three ZurichMOVE Sensors was mounted on participants’ wheelchairs, one on the main frame, and one on each wheel to allow the assessment of motion and turning.

One sensor was rigidly mounted on the spokes of each wheel and one was fixed to the frame below the seat using custom-made, durable adaptors (Figure 1). Primarily, the gyroscopic data from the wheels was used to derive kinematic parameters, such as velocity or wheeled distance, while the sensor on the frame was used to estimate the wheelchair’s orientation. This information allowed the detection of redundant situations, e.g., when the wheelchair was transported in the boot of a car, or when participants used a wheelchair tractor. Here, the movement detected by the sensors was not considered of interest as participants were not actively propelling. In addition to the assessment of movement and turning, participants were asked to report daily routines in a questionnaire for each day of the measurement. All phases of movement data that were suspected to be redundant were crosschecked with the information from the questionnaires and excluded from the analysis.

### Data Analysis

Data processing was performed using MATLAB 2020a (MathWorks, Natick, MA, United States). Before further calculations, the orientation of the sensors was corrected. For the sensors on the spokes, time periods with a straight-forward wheeling movement (difference between left and right wheel velocity smaller than 0.05 m/s) with a duration longer than one second were identified in order to rotate the sensor’s coordinate systems such that the respective *z*-axis coincided with the rotation axis, z_rot_, of the wheelchair wheels. For the sensor on the frame, the time periods where the wheelchair was at rest (left and right angular velocity smaller than 1.15°/s) were used to align the *z*-axis of the sensor with the direction of the gravitational acceleration, z_g_ (Figure 1). These realignments of the sensor coordinate systems have been performed for all the identified time periods.

To identify and describe turns and turning patterns, a two-step process was undertaken: firstly, only phases in which the wheelchair was moving were considered of interest, and secondly, a reconstructed movement path served as a basis to estimate turning radii and angles.

#### Wheelchair Movement

Phases of wheelchair movement were identified, using the definition of moving windows by Popp and co-workers (Popp et al., 2016): The wheelchair was considered to be moving if a threshold of 0.4°/s was recorded by at least one sensor on the wheels, otherwise the wheelchair was at rest. For a valid moving window, the gyroscopic data had to reach 10°/s at least once, while the wheel had to rotate by at least 80°. The minimum duration of a moving window was 2 s and if two consecutive moving windows were separated less than 2 s, they were merged. All classified moving windows were used for further analysis.

**FIGURE 2:**
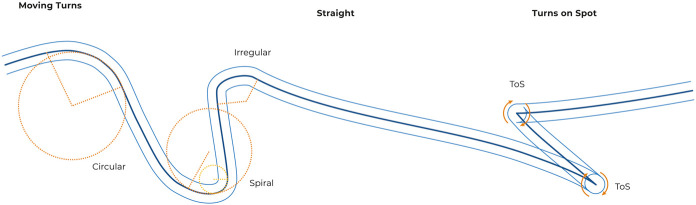
Examples of classified segments: Moving windows were divided into straight sections, turns-on-the-spot and moving turns whereby the latter were further subclassified as regular, spiral or irregular turns based on their circularity.

To derive wheeled distances and directions, angular velocities of both wheels were multiplied with the wheel’s radius and then integrated. Knowing the wheelchair’s track width allowed the reconstruction of the wheeled path in a 2-dimensional space over time to provide a basis for identifying turns (Figure 2).

#### Turn Identification and Classification

To determine start- and end-points of a turn during the recognised moving windows, a distinction between moving straight-forward and turning was made. Segments in which the wheel velocities differed by less than 0.05 m/s were considered as straight. Turns were investigated in the remaining time periods.

The signs of the wheel velocities enabled a differentiation between “moving turns” and “turns-on-the-spot”. The latter were characterised by one wheel moving forward while the other moved backwards, and were detected by opposite signs in the directional data. Assuming that no notable distance was covered for this type of turn, the turning radius was set to zero. All identified turns-on-the-spot were taken into account if all of the following conditions were met:

• The duration of a turn-on-the-spot was greater than 0.5 s.
• The mean angular velocity of a turn-on-the-spot was greater than 20°/s.
• The turning angle of a turn-on-the-spot is at least 45°.

Two consecutive turns-on-the-spot were merged if they were separated by less than 0.3 s. Moving turns were then analysed based on the previously reconstructed wheeled path. Here, turning radius was approximated for each segment using least squares fitting according to Pratt (Pratt, 1987) (Figure 2). Moving turns were taken into account if all of the following conditions were met:

• The duration of a moving turn was greater than 0.5 s.
• The mean velocity during a moving turn was greater than 0.12 m/s.
• The wheeled distance of a moving turn was greater than 20 cm.
• The turning angle of a moving turn was at least 15°.

Consecutive moving turns were merged if they were separated by less than 0.3 s, or the wheeled distance in between was less than 20 cm. Furthermore, moving turns with a radius greater than 10 m were considered as straight-forward movements. All parameters were chosen based on repeated test measurements where known movement paths like rectangular trajectories, circles, figure-8s and straight lines with turns-on-the-spot at their ends where wheeled. This helped finding definitions that result in plausible distinctions between different types of movement (moving turns, turns-on-the-spot, straightforward).

To verify circularity, the normalised root-mean-square error (NRMSE) of the turning radius was calculated for each moving turn. Turns with NRMSE <4% were classified as “regular turns”. Non–circular turns with NRMSE >4% were further analysed. Here, the first third and the last third of the moving turn were again approximated with the Pratt method in order to examine whether the turning radius was increasing or decreasing. If both new fitted circles had an NRMSE <4%, then the ratio of the two radii was calculated. Turns with a ratio smaller than 0.5 or higher than 2 were classified as “spiral turn” (Figure 2). All remaining turns were classified as “irregular turns”. Spiral and irregular turns were not assigned with a turning radius.

#### Turn Properties

Turns where the absolute velocity of the right wheel was greater than that of the left wheel were categorised as left turns, and the remaining as right turns. Moving turns were further classified as forward or backward turns based on the signs of the wheel velocities. For all moving turns, turning distance was calculated by integrating the center of mass velocity of the wheelchair. For regular turns, turning angles and radii were derived from the fitted circle. For turns-on-the-spot, the angular velocity in the direction of the axis of rotation (z_g_) from the sensor on the frame was used for calculation.

#### Movement Patterns and Analysed Parameters

To recognise typical turning sequences, actions one second before and after every turn were analysed. Actions were categorised as: another moving turn, another turn-on-the-spot, straightforward movement, or standstill.

To describe moving windows, the following parameters were investigated: duration, distance, mean velocity, included number of turns, and separation between two consecutive moving windows. For turns, the set of parameters included radius, angle, duration, distance, mean velocity, velocity at start, velocity at the end, and action 1 s before and after the turn. To describe the overall wheelchair usage in terms of moving time, covered distance, and number of moving windows, only complete days of measurement were considered.

#### Error Analysis

To estimate the influence of the error of the IMU sensors on parameters of interest, an error analysis was performed. Based on the specifications of the used IMU sensors, the error of the gyroscopic data can reach 2°/s. This maximum error was added to the data of one sensor on the spokes as well as to the measurements of the sensor on the frame to gauge maximum impact on the evaluated parameters. Analysis was performed once with and once without consideration of this maximum error for all measurements. Absolute and percentual differences of all received parameters were calculated.

## Results

### Participant Characteristics

Fourteen adults who use active wheelchairs for mobility participated in this study with sensors tracking their activities for a mean duration of 163 ± 12 h (range 135–176 h). The majority of participants had a thoracic spinal cord injury, although one individual with tetraplegia, one with cerebral palsy, and one with knee disarticulation were also included. Additional descriptions of all participant characteristics are presented in Table 1.

**TABLE 1:** Characteristics of included participants (N = 14).

Characteristic	n or mean ± SD (range)
Age (years)	37 ± 12 (18–61)
Time in Wheelchair (years)	13 ± 8 (3–27)
Sex	
Male	12
Female	2
Handedness	
Right	13
Left	1
Injury Level	
C6–C7	1
T1–T12	11
Other (CT, Knee Disarticulation)	2

### Error Analysis

The expected maximum measurement error was added to the individual sensor values in order to analyse the maximum offset of parameters of interest and assess their reliability. A comparison between the directly calculated results with artificially erroneous ones over the entire data collection yielded the error values given in Table 2. For moving turns, the addition of 2°/s to the gyroscope values of the wheel on the side of the turn resulted in wider turns, whereas on the other side resulted in tighter turns. The mean velocity depends directly on the angular velocity and resulted in a maximum error of 0.5 cm/s.

**TABLE 2:** Values from error analysis in units and percentage.

Metric	Moving turns						Turns on spot			
	Radius		Angle		Distance		Angle		Mean velocity	
	cm	%	°	%	cm	%	°	%	°/s	%
Mean	8.36	5.21	2.38	5.14	1.08	1.25	3.76	4.13	2.03	4.13
SD	1.04	0.74	0.47	0.72	0.15	0.18	0.43	0.56	0.01	0.56
RMSE	11.17	5.11	1.08	4.01	3.76	1.22	3.48	3.74	1.72	3.74

### Moving Windows

The datasets contained a total of 31′410 moving windows. The mean number of daily moving windows was 331 ± 101. Daily moving time was 71 ± 24 min on average, whereby a mean daily distance of 3.10 ± 1.44 km was covered. A large proportion of moving windows were rather short, with a per person median duration averaging 7.0 ± 0.9 s and a median distance of 2.3 ± 0.6 m. Mean median velocity was 0.35 ± 0.05 m/s.

**FIGURE 3:**
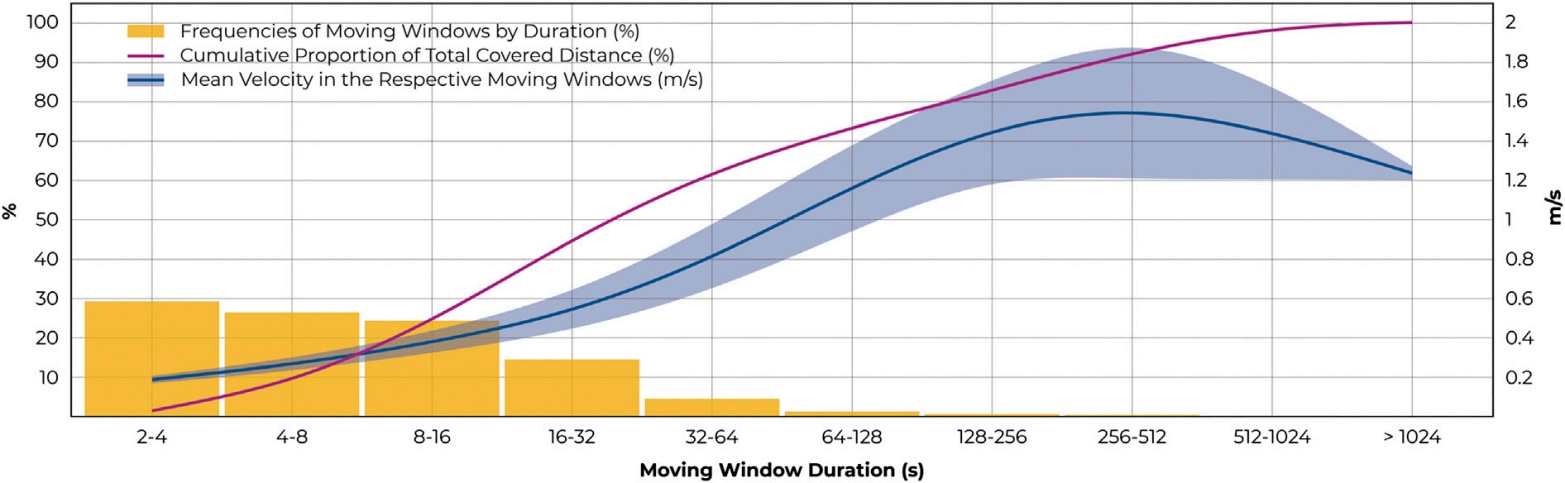
Relationships between duration, distance, and velocity of moving windows. The distribution of moving window durations is given in the yellow bars while the purple line indicates the cumulative proportion of the total covered distance. The mean velocities and standard deviations in the respective data bins are shown in blue.

Probing moving windows by duration, 64 ± 5% were found to be shorter than 10 s (Figure 3). During these sections, a mean distance of 1.5 ± 0.2 m was covered at a mean velocity of 0.24 ± 0.02 m/s. Cumulating the 93 ± 2.7% of moving windows that were shorter than 30 s, a mean distance of 3.90 ± 0.70 m and a mean velocity of 0.31 ± 0.04 m/s was found. Only 2.0 ± 1.2% of moving windows were longer than 60 s, associated with a mean distance of 197.7 ± 92.0 m and a mean velocity of 1.23 ± 0.24 m/s. Despite the infrequent occurrence of longer, continuous movement, 40.0 ± 21.0% of the total distance covered were wheeled in moving windows of longer than 60 s. In other words, during the longest 20% of moving windows, participants covered 74.3 ± 8.5% of total distances, almost directly following the Pareto principle.

Consecutive moving windows were separated by a median time of 16 s, and a mean time of 151 s 78% were separated by less than 60 s, 57% by less than 20 s.

### Turn Characteristics

A total of 19′673 turns-on-the-spot and 66′829 moving turns was detected in the collected data. Among the latter, 97.2% were classified as regular and 1.9% as spiral. The remaining 658 irregular turns (0.9%) were excluded from the analysis.

No participant showed a clear preference with regards to turning direction, resulting in almost equal number of left and right turns in both moving turns and turns-on-the-spot. The majority of moving turns, 89.1 ± 4.3% were wheeled in forward direction.

**FIGURE 4:**
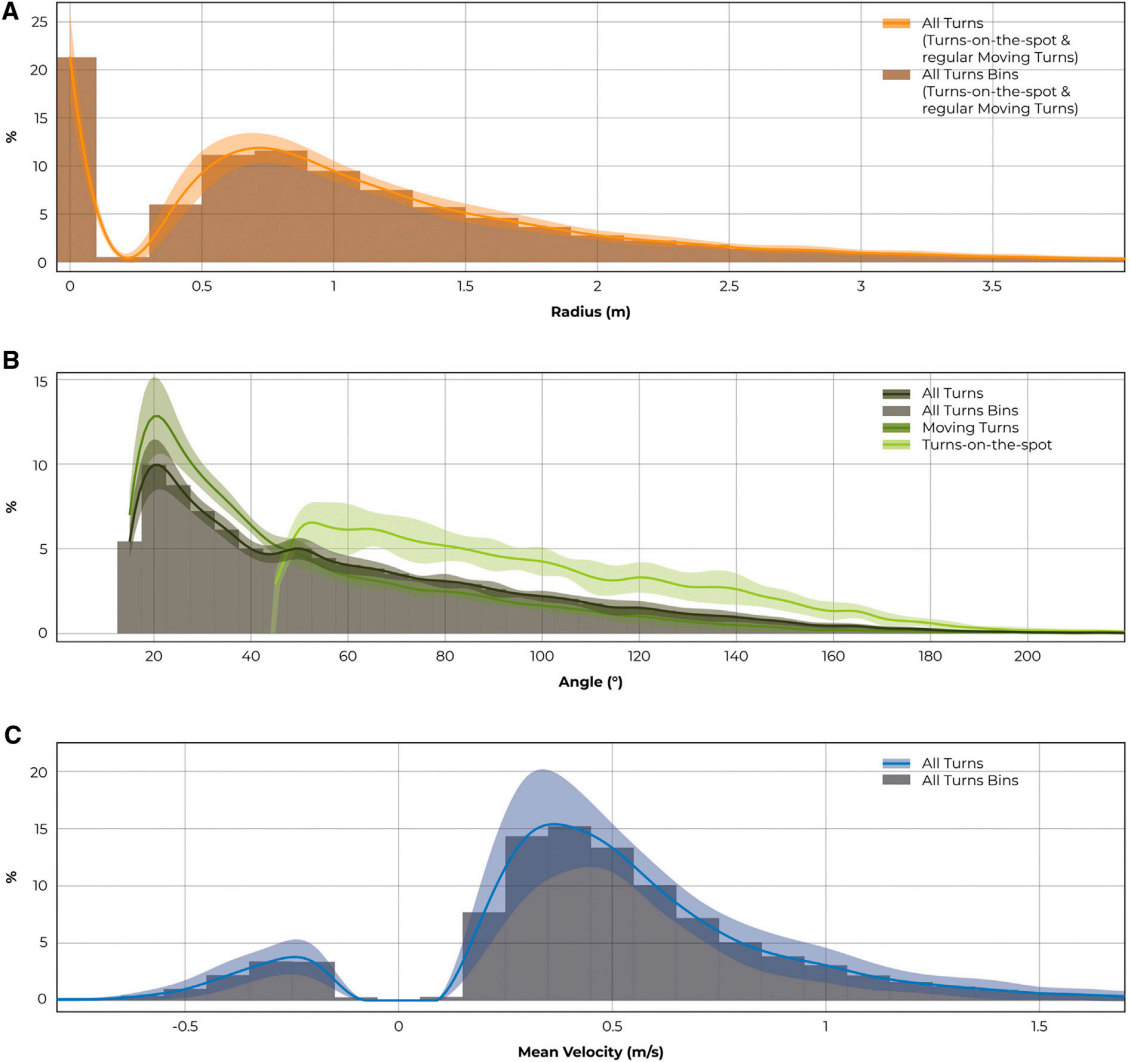
Distributions of **(A)** turning radii, **(B)** turning angles and **(C)** mean velocities of turns are shown. Shaded areas indicate the respective standard deviations. Histograms are given to represent the respective sizes of analysis bins of 0.2 m, 5°, and 0.1 m/s.

Overall, the median turning radius was found to be 1.09 ± 0.12 m, whereby 90% of the radii were smaller than 3.33 m (Figure 4A). The median angle for moving turns was 39.1 ± 4.2°, and 89.5 ± 8.1° for turns-on-the-spot. 90% of turning angles were smaller than 111.2 ± 19.0° among moving turns and 148.1 ± 7.6° for turns-on-the-spot (Figure 4B).

Median mean velocity for moving turns was found to be 0.49 ± 0.09 m/s, while backward turns were slower than turns wheeled forward (Figure 4C). Durations of all turns were rather short. Moving turns took a median duration of 2 s while wheeling a distance of 0.8 m. A similar median duration was found for turns-on-the-spot (Table 3).

**TABLE 3:** Characteristics of all identified turns.

Turning Metric	Mean	± SD	Median (IQR)
Radius (m)			
Moving Turn	1.39	±0.20	1.09 (0.73–1.72)
Angle (°)			
Moving Turn	51	±5	39 (24–67)
Turn-on-the spot	97	±7	89 (65–119)
Duration (s)			
Moving Turn	2.1	±0.3	1.8 (1.2–2.6)
Turn-on-the-spot	1.9	±0.2	1.7 (1.3–2.3)
Distance (m)			
Moving Turn	1.17	±0.18	0.84 (0.46–1.55)
Mean Velocity (m/s)			
Moving Turn	0.57	±0.14	0.49 (0.34–0.71)

**FIGURE 5:**
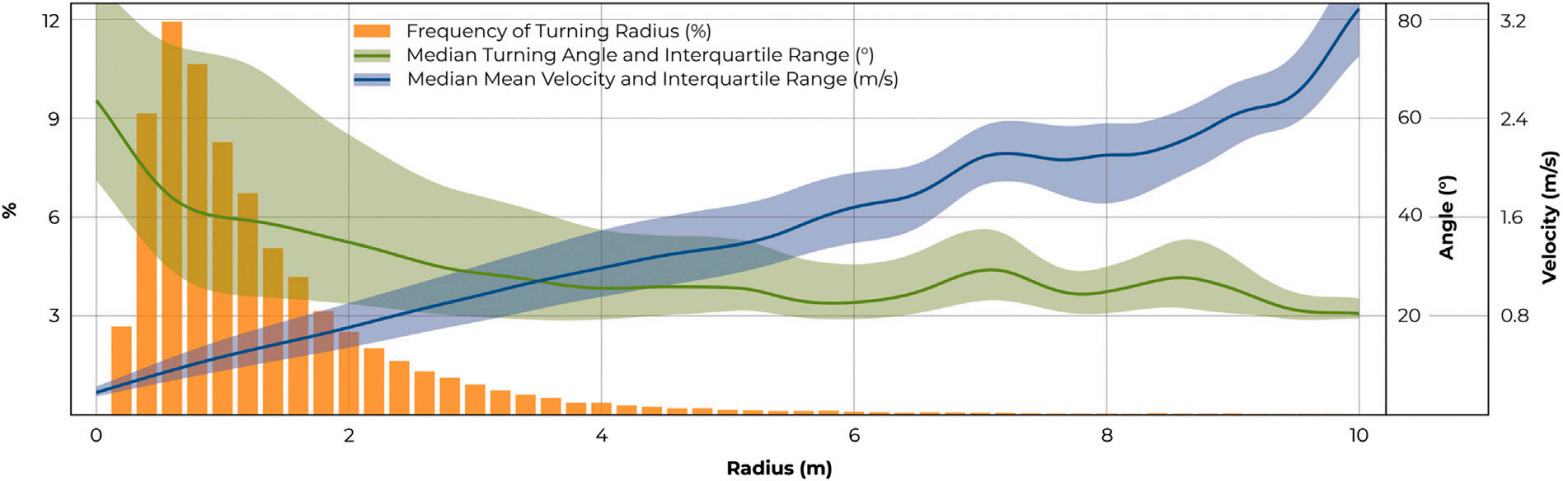
Relationships between turning radii, angles, and velocities for regular moving turns. Records were discretised according to turning radius in steps of 0.2 m. Frequencies are shown in orange with respective median turning angles overlayed in green and median mean velocity (for moving turns) in blue. Shaded areas indicate the interquartile ranges.

Turning with a radius of 0.6–0.8 m was found to be most frequent (Figure 5). In this range, median mean velocity was 0.36 m/s and median turning angle was 40°. Turns with smaller radii were associated with slower speeds and higher turning angles (for turns with a radius of 0.2–0.4 m, a median velocity of 0.24 m/s and a median angle of 61° was observed) whereas larger bends coincided with higher velocities and smaller angles (turns with a radius of 6.0–6.2 m produced a median velocity of 1.71 m/s and median angle of 23°). Scrutinising extremes in the data confirmed this trend: Highest velocities were found among turns with highest radii, as, for example, velocities greater than 2 m/s only occurred during turns with radii greater than 4 m. Similarly, highest turning angles were detected for turns with the smallest radii. For instance, turning angles of more than 120° only occurred at radii of less than 2.4 m.

### Movement Patterns

During the week-long measurement period, participants wheeled a mean 897 ± 328 turns per day, whereby moving turns accounted for 77 ± 5% of all recognised turns (Table 4).

**TABLE 4:** Frequencies of moving turns and turns-on-the-spot reported per day and moving window. Time and Distance intervals between turns were calculated during time spent moving.

Metric	Mean ± SD
Turns per Day	913 ± 274
Moving Turns	706 ± 212
turns-on-the-spot	207 ± 66
Turns per Moving Window	3.1 ± 0.5
Moving Turns	2.4 ± 0.5
turns-on-the-spot	0.7 ± 0.1
Moving Distance Interval (m)	3.6 ± 1.5
Moving Turns	4.6 ± 1.9
turns-on-the-spot	17.0 ± 8.3
Moving Time Interval (s)	4.9 ± 1.0
Moving Turns	6.2 ± 1.4
turns-on-the-spot	22.8 ± 6.7

About 30% of all moving windows started with a turn-on-the-spot. 12% of all windows contained neither a moving turn nor a turn-on-the-spot. In the remaining 88%, a mean of 3.1 turns per moving window, composed of 2.4 moving turns and 0.7 turns-on-the-spot, were detected. During moving windows, a turn was wheeled every 4.9 s, with an interjacent mean distance of 3.6 m. More specifically, every 6.2 s a moving turn, and every 22.8 s a turn-on-the-spot was identified, with a mean distance interval of 4.6 m, and 17.0 m, respectively.

**FIGURE 6:**
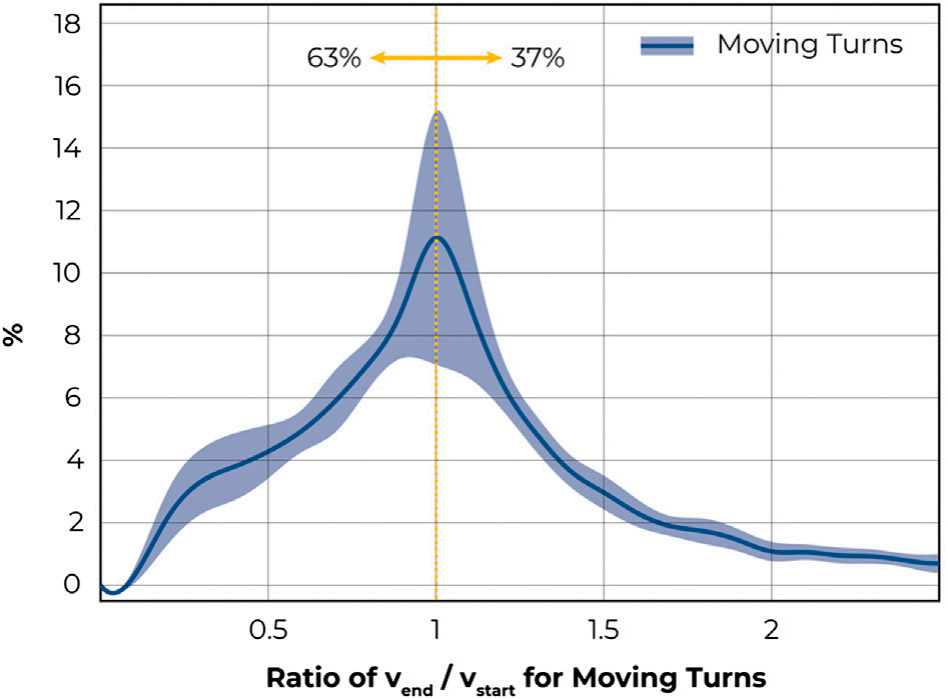
Graph showing ratio between initial and final velocity of moving turns. Only moving turns where the wheelchair was considered to be moving before and after the turn were included. A ratio greater than 1 indicates an increase in velocity over the course of the turn. 63% of turns were associated with a decrease in velocity.

A comparison between initial and final velocity of moving turns showed that during 63 ± 3%, velocity was lost during the turn (Figure 6). This assessment accounted for turns that started from or ended in a standstill, which were excluded from the analysis.

In order to describe typical movement sequences, actions 1 s before and after each turn were assessed. For actions before moving turns, a straightforward movement was found to be the most common, accounting for 41% of the cases. The second most frequent action was another moving turn (25%), a standstill (19%), or turn-on-the-spot (15%). The majority of turns-on-the-spot started from rest (58%) or occurred immediately after a moving turn (29%). Activities including a turn-on-the-spot following a straightforward movement, or another turn-on-the-spot, appeared less frequently (12%, and 1%, respectively).

After a moving turn, participants continued with a straightforward movement in about 47% of cases, wheeled a subsequent moving turn in 25%, and stopped directly afterwards in 20%. Another turn-on-the-spot was least frequent with 8%. More than half of all turns-on-the-spot were directly followed by a moving turn (55%), while the wheelchair was stopped in 25%, or superseded by a straightforward movement in about 19% of cases. Turns-on-the-spot following each other were found to be infrequent (<1%) (Table 5).

**TABLE 5:** Identified action 1 s before and after a turn.

Action	Moving turn	Turn-on-the-spot
	Mean ± SD	Mean ± SD
Before (%)		
Moving Turn	24.6 ± 7.5	28.9 ± 6.0
Turn-on-the-spot	15.4 ± 3.3	0.9 ± 0.5
Straightforward Movement	41.1 ± 4.9	11.9 ± 3.1
Standstill	18.9 ± 3.7	58.3 ± 5.4
After (%)		
Moving Turn		54.5 ± 8.1
Turn-on-the-spot	8.1 ± 1.9	
Straightforward Movement	47.1 ± 7.3	19.3 ± 3.9
Standstill	20.2 ± 3.4	25.3 ± 6.4

All percentage values were calculated using total number of identified moving turns or turns-on-the-spot

## Discussion

As enablers of mobility, participation and health, wheelchairs critically shape users’ actions - knowledge about which can, in turn, guide the design and customisation of future assistive technologies. Towards developing novel wheelchair-user-interfaces, this study characterises the turning behaviour of wheelchair users during normal functional activities of daily living. With about 900 turns per day or a turn every 3.6 m, changing and adjusting direction is fundamental in wheelchair usage. A median turning radius of 1.09 m and a median angle of 39° for moving turns suggest that this is typically achieved in sharp corners rather than large bends. Similarly, turns-on-the-spot account for about a quarter of recognised activities and are typically associated with high angles (in the range of 60°–120°), with a majority starting from a standstill. In summary, manoeuvring manual wheelchairs in daily life is best described as a concatenation of orienting turns-on-the-spot, short straight sections, and tight, corrective moving turns.

Adding to a body of research on the study of daily activities using wearable sensors, wheelchairs of 14 active wheelchair users were instrumented with a set of 3 IMUs for measurement of an entire week. From these participants, a total of 2,302 h and 334 km of wheelchair movement distributed over 31′410 moving windows that contained 86′565 turns was analysed within this study. Error analysis substantiated that a valid method was chosen to describe the kinematics of turning wheelchairs in daily life. Maximum theoretical errors were small, 5% (NRMSE) or less for all analysed parameters, implying that the analysis results could only been marginally affected by possible inaccuracies in the measured IMU sensor data.

The measured mean wheelchair velocity during moving windows, turns, and straightforward movements of 0.35 m/s, 0.57 m/s, and 0.48 m/s, respectively, are comparable with previous literature ranging from 0.2 to 0.8 m/s (Tolerico et al., 2007; Cooper et al., 2008; Karmarkar et al., 2010; Oyster et al., 2011; Sonenblum et al., 2012a). The mean daily wheeled distance of 3.10 km in this study first appears high compared to former reported values ranging from 1.87 to 2.46 km per day (Tolerico et al., 2007; Oyster et al., 2011; Sonenblum et al., 2012c). Tolerico et al. (Tolerico et al., 2007) stated that employed users wheeled a higher daily distance compared to unemployed users as excluding unemployed subjects from their analysis raised the mean daily covered distance from 2.46 to 3.40 km. In the present study, 12 of the 14 included subjects indicated work related activities in their completed questionnaires. In other studies, higher proportions of unemployed participants were included, ranging from 36% in Sonenblum and others’ work (Sonenblum et al., 2012c) to 64% in the publication by Oyster and co-workers (Oyster et al., 2011). This might further explain why the average daily recorded time moving (71 min) was higher in our case than in the two previous studies (47 and 58 min, respectively), though cultural differences or seasonal aspects should also be considered.

For a general description of activity, Popp and others’ definition of moving windows was used (Popp et al., 2016) instead of Sonenblum and co-workers’ bouts of mobility (Sonenblum et al., 2012c). The latter refer to moving segments of a minimum duration of 5 s, however, 37% of the detected moving windows observed in our study were shorter than 5 s. Assuming that such short manoeuvres represent a key requirement in terms of the flexibility of a wheelchair, they contain valuable information for an extensive description of typical movement patterns. If only moving windows longer than 5 s were considered, mean and median velocities would increase to 0.44 m/s and 0.39 m/s, which are comparable to Sonenblum and co-workers’ results of 0.48 m/s and 0.43 m/s, respectively.

During the measurement periods, an average of 331 moving windows per day were detected. Echoing other authors’ findings (Sonenblum et al., 2012b), most of these were rather short and slow with a median duration of around 7 s and a median distance of 2.3 m, which is just slightly more than one full wheel revolution. Despite the low frequency of longer moving windows, they appear to be integral to user’s actions: almost 75% of the total distances were covered during the longest 20% of moving windows–that is, longer than approximately 16 s. During such longer, continuous movements, mean velocities tended to be higher than during short, typically just a few seconds long movements. Still, these results suggest that starting and stopping is an important characteristic of the wheeling habits of manual wheelchair users during their daily routine. Propulsion costs to accelerate a wheelchair from standstill are higher compared to maintaining a constant velocity while wheeling (Koontz et al., 2005). To improve efficiency and reduce the stress building up in the upper extremities of users, minimising these forces–for example by reducing the overall weight of the wheelchair or optimising push angles–could be an important requirement for future wheelchair designs. Equally, promoting fewer, but longer and more continuous moving windows instead of stop-and-go wheelchair usage would help mitigating the risk of shoulder injuries.

On average, a moving window contained about 3 turns, most of which were characterised by small turning radii in the range of up to twice the wheelchairs’ track widths as well as small turning angles of less than 90°. Instead of turning in large curves, wheelchair users seemed to prefer to move larger parts of a distance in a straightforward movement and adjust direction in short, tight turns or–in other words–it appears they favour a polygon over continuous circumscribing circles. Literature that helps explaining these findings is extremely limited. In 1990, Reid and co-workers (Reid et al., 1990) attempted to measure the physiological energy cost of steering by comparing wheelchair usage in 3 different tracks against straightforward propulsion on a treadmill. They concluded that the effort for steering contributes significantly to the overall energy cost of wheelchair propulsion, particularly at higher speeds. In consequence, it would seem natural that wheelchair users try to minimise the number of turns in their activities. However, spatial constraints or desired destinations might not leave users a choice over whether or not to adjust direction but rather when and where. A recent statistical modelling approach by Misch et al. showed that propulsion costs for turns-on-the-spot (zero–radius turns) are lower compared to “fixed wheel turns” (one wheel remaining still resulting in a turning radius of half the wheelchair’s track width) (Misch et al., 2020), i.e., simultaneous pushing and braking requires more effort than symmetrically pushing. Consequently, major changes of direction with turns-on-the-spot are energetically preferable, which might explain why participants of this study tended to complete larger turning angles by turns on-the-spot. Moving turns with rather large radii often involve pushing with one hand and braking with the other at the same time. This process is complex, and as previously discussed, inefficient. Propulsion inefficiency can be described by the velocity loss, which might explain that 63% of moving turns, for which the wheelchair was moving both before and after the turn, ended slower than they started.

Assuming that wheelchair users intuitively optimise energy consumption to at least some degree, both the overall high frequency of turns and the predominance of sharp moving turns with small angles appear to be reasonable consequences of the conventional wheelchair configuration that, at least during movement, requires users to break on one side in order to change direction. The large proportion of turns-on-the-spot further supports this argument, especially given frequent movement patterns: almost 60% of turns-on-the-spot started from standstill and about 25% ended a moving window. Roughly a third of moving windows directly started with a turn-on-the-spot. A typical technique of reaching a desired destination appears to be to first align the wheelchair’s orientation with a turn-on-the-spot, then start moving in the desired direction and later adjust the direction with short moving turns as needed. This explanation is further supported by the finding that moving turns often occur during movement or between straightforward segments.

The relevance of these results is two-fold: firstly, they highlight the importance of good maneuverability of wheelchairs. The ability to change direction quickly and fluently seems to be essential. Secondly, the kinematics of manoeuvres in wheelchairs are testimony to the conventional wheelchair-user-interaction principle that offers great flexibility thanks to the usually small and light castor front wheels, but on the other hand, this arrangement is inefficient for the precise control of direction. The present study provides a unique insight into wheelchair users’ daily routines, gearing towards the development of novel, steering wheelchairs. Future wheelchair designs will need to offer effortless turning-on-the-spot, and allow small turning radii together with controlled and efficient turning at higher speeds.

### Limitations and Outlook

One limitation was that only 14 wheelchair users participated in this study. Even though some trends were found, inter-subject variability was relatively large. In addition, 85% of participants in this study had a spinal cord dysfunction. The observed results are likely to differ if more subjects with other walking disabilities were included. Next, long-term data collection in daily live can be influenced by many unknown factors such as sensors getting caught or subjects manipulating their position. Even though several measures to prevent sensor misalignment were taken, slight changes to the sensors positions relative to participants’ wheelchairs could have had a marginal influence on the study results. Equally, this study’s outcomes are the results of chosen methods. Especially the definitions of turns, turns-on-the-spot and straightforward movement clearly influenced the central findings. To avoid arbitrary choices, these central parameters were carefully developed in repeated test measurements. Furthermore, some participants reported that they predominantly stayed at home during the measurement period due to the outbreak of COVID-19 and could have moved further and more frequently under different circumstances. The overall distances travelled however still appeared comparable to or even exceeded those reported in previous studies. These limitations imply that a generalisation about overall wheelchair use is difficult, and further studies with a higher number of participants of greater diversity would be needed in order to obtain more conclusive results.

This study aimed at describing manoeuvres of wheelchair users in general and did not consider any potentially stratifying factors such as the types of activities, users’ experience and skills or specific wheelchair models. Such comparisons could be the target of further studies. Similarly, the investigation should be repeated if wheelchairs with new steering concepts become available. Potential differences in movement patterns between different wheelchair technologies would highlight the extent to which assistive device mechanisms are able to influence and support users’ behaviour, and hopefully also decrease the risk of injuries and developing pain due to overuse.

It is important to note that the methods developed in this study might also be useful in other fields. Therapists could use similar techniques to monitor patients’ activities in order to tailor treatments and training more specifically to each individual’s skills and abilities–especially as different turning techniques are among the most essential wheelchair skills (Hosseini et al., 2012). In court sports like rugby, basketball or tennis, such approaches for identifying and quantifying turns might be an insightful addition to methods used for the assessment of athletes’ performance with the goal of improving training plans or athlete classification (van der Slikke et al., 2015).

## Conclusion

Knowledge about reoccurring patterns in manual wheelchair movement during everyday life can critically contribute to improvements in rehabilitation and related technologies. Analysis of moving windows, turn characteristics, and typical movement patterns in this study provide deeper insight into how wheelchairs are used. Our data suggest that initiating movements, stopping, covering short distances, and manoeuvring in small circles dominate the daily wheelchair usage. These movements are associated with a change in inertia and requires effort from the user. Future wheelchair mechanisms should reflect this by minimising such energy costs to improve efficiency, and develop more ergonomic, energy-efficient, and user-friendly wheelchairs. More research is needed to investigate if the turning behaviour directly depends on the wheelchair type used, or differs significantly between different activities, for example by differentiating indoor and outdoor use. If movement patterns differ considerably, future wheelchairs should offer adaptive mechanisms to switch to the appropriate environment.

## Data Availability

The raw data supporting the conclusions of this article will be made available by the authors, without undue reservation.
